# Spread Spectrum Image Watermarking Through Latent Diffusion Model

**DOI:** 10.3390/e27040428

**Published:** 2025-04-15

**Authors:** Hongfei Wu, Xiaodan Lin, Gewei Tan

**Affiliations:** School of Information Science and Engineering, Huaqiao University, Xiamen 361021, China; 23014082023@stu.hqu.edu.cn (H.W.); tangewei70@163.com (G.T.)

**Keywords:** image watermarking, spread spectrum, latent diffusion model, information hiding

## Abstract

The rapid development of diffusion models in image generation and processing has led to significant security concerns. Diffusion models are capable of producing highly realistic images that are indistinguishable from real ones. Although deploying a watermarking system can be a countermeasure to verify the ownership or the origin of images, the regeneration attacks arising from diffusion models can easily remove the embedded watermark from the images, without compromising their perceptual quality. Previous watermarking methods that hide watermark information in the carrier image are vulnerable to these newly emergent attacks. To address these challenges, we propose a robust and traceable watermark framework based on the latent diffusion model, where the spread-spectrum watermark is coupled with the diffusion noise to ensure its security and imperceptibility. Since the diffusion model is trained to reduce information entropy from disordered data to restore its true distribution, the transparency of the hidden watermark is guaranteed. Benefiting from the spread spectrum strategy, the decoder structure is no longer needed for watermark extraction, greatly alleviating the training overhead. Additionally, the robustness and transparency are easily controlled by a strength factor, whose operating range is studied in this work. Experimental results demonstrate that our method performs not only against common attacks, but also against regeneration attacks and semantic-based image editing.

## 1. Introduction

Digital image watermarking enables information to be added to an image to protect the copyright of the image without compromising its visual quality. To achieve effective watermarking, the watermark should be recovered at the receiver side even when suffering from various distortions like JPEG compression, blurring, etc.

Traditional methods typically embed watermarks in the spatial domain, like the LSB embedding [1], or modify the coefficients in the transform domain using transformations such as discrete Fourier transform (DFT), discrete Cosine transform (DCT), and discrete Wavelet transform (DWT) [2,3]. As deep learning advances, the paradigm of image watermarking shifts from explicit spatial or transform domain to the implicit latent space of high dimensions. An end-to-end encoder–decoder framework was initially proposed in [4]. To tackle the robustness issue, researchers optimize the design of the noise layer via data augmentation. Tancik et al. [5] embed an invisible hyperlink to the images, utilizing the UNet style structure [6] to encode and decode the secret hyperlink information. The authors add various differential operations, approximating the print-shooting distortions in the noise layer to enhance its robustness. To counter the compression attack, the authors in [7] propose a mini-batch scheme, which changes the noise layer from real JPEG, simulated JPEG, and noise-free ones. Furthermore, Wang et al. [8] introduced an adaptive network which optimizes the embedding factor. A decoupled noise layer against screen-capture attacks is proposed in [9], where style transfer is employed to mimic channel distortions and aid in network training. Fernandez et al. [10] integrated the watermark into the latent space of a neural network pre-trained via self-supervised learning, effectively building feature space to hide the watermark. In recent years, with the development of diffusion models, researchers have discovered their potential in the field of watermarking. Zhao et al. [11] implanted watermarks into the training images to achieve copyright protection when generating images with diffusion models. By fine-tuning the latent decoder in the latent diffusion model, embedding messages in generated images was achieved in [12]. A stable diffusion based image watermark pipeline is proposed in [13], which embeds a tree-ring pattern [14] in the Fourier domain of the latent space. Tan et al. [15] achieved perceptual image watermarking by embedding the watermark in the low-level feature space generated by diffusion models.

**Figure 1:**
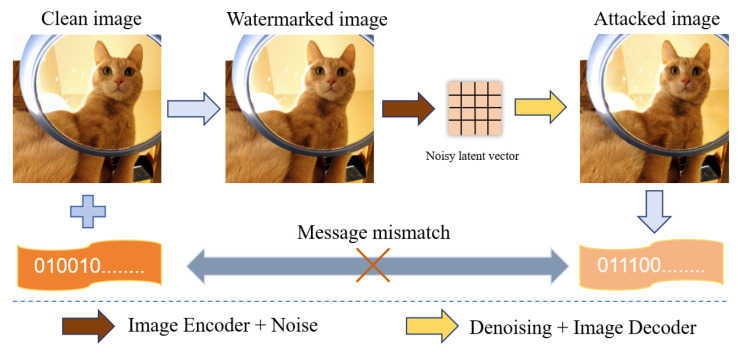
Image regeneration attack.

In spite of the merits of the new modality of image watermarking via deep learning frameworks, the rapid development of generative models also raises new security concerns. A recent study in [16] showed that processing images with the latent diffusion model can remove watermarks from most existing methods. As shown in Figure 1, this regeneration attack can inject noise into the latent space of images to disrupt the original distribution, and then denoise it to regenerate images. This regeneration process can remove watermarks while preserving the fidelity of images. Additionally, text-to-image diffusion models can easily edit images with prompts, resulting in the unavailability of the embedded watermarks. However, most watermarking approaches did not take the regeneration attack and local editing into account, resulting in the vulnerability of the watermarking systems.

Since the regeneration attack and image editing are launched with the diffusion model, the latent representation of images integrated with the diffusion model can be an ideal place to hold the watermark. The spread spectrum watermark that draws on spread spectrum technology from communications has been proven to be a success in traditional watermarking schemes [17], as it spreads the watermark signal over a wide range of frequency bands, guaranteeing its security and imperceptibility. Therefore, it is employed to enhance the security and robustness of the watermark. Our contributions can be summarized as follows:

- The proposed scheme embeds the watermark information in a spread spectrum manner. It does not require a pre-training embedding network or a decoder structure, and therefore, the network is shown to be more lightweight.
- The noisy feature representation of the latent vector is explored to hide the watermark message. Benefiting from the reversible property of diffusion models, the watermarked image can be produced without compromising the perceptual quality. Additionally, the VAE encoder acts as a robust feature extractor, which also provides a sweet spot to accommodate the watermark. Furthermore, the diffusion operated on the lower dimensional vector yielded by the VAE-encoder is computationally efficient.
- Experimental results show that our framework performs robustly under common attacks like JPEG compression, brightness adjustment, and blurring. More importantly, it is resistant to image regeneration and image editing.

## 2. Method

**Figure 2:**
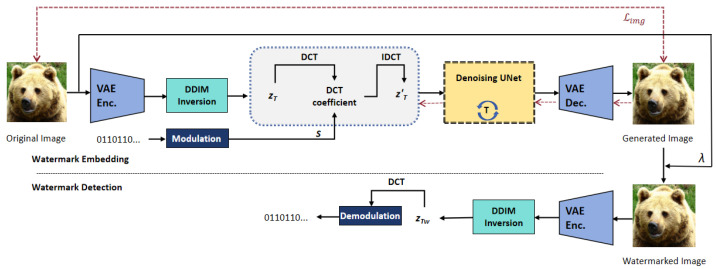
The proposed framework.

Figure 2 shows the framework of the proposed method. The clean image is fed into the pipeline to obtain noisy latent space. Next, we embed modulated spread spectrum watermark into the noisy latent vectors. Finally, the denoising network and the decoder for the latent vector are used to generate watermarked images. Unlike the existing deep learning-based watermarking schemes, which typically require a decoder network to restore the watermark, blind detection is achieved in this work by calculating the correlation between the watermark patterns and the feature representation vector in noisy latent space.

### 2.1. Diffusion and Inversion

The diffusion process in DDPM [18] adds Gaussian noise to the original data in T-steps through a Markov chain to obtain a noisy image $x_{T}$ as

(1) $$q\left(x_{t}|x_{t-1}\right)=N\left(x_{t};\sqrt{1-\beta_{t}}x_{t-1},\beta_{t}I\right),$$

where $\beta_{t}\in \left(0,1\right)$ is the variance at the step of t and t∈(0,1,…,T−1). In the reverse process, the generation requires a noise predictor with which the noise is removed to restore the images. However, this diffusion process is stochastic and is therefore detrimental to the reconstruction of watermarked images. In contrast, DDIM inversion [19] implicitly adds predicted noise at each diffusion time step and outputs the final distribution as structured noise. This process can be defined as

(2) $${\hat{x}}_{0}^{t}=\frac{x_{t}-\sqrt{1-{\bar{\alpha }}_{t}}\epsilon_{\theta }\left(x_{t}\right)}{\sqrt{{\bar{\alpha }}_{t}}},$$

(3) $$x_{t+1}=\sqrt{{\bar{\alpha }}_{t+1}}{\hat{x}}_{0}^{t}+\sqrt{1-{\bar{\alpha }}_{t+1}}\epsilon_{\theta }\left(x_{t}\right),$$

where ${\bar{\alpha }}_{t}=\prod_{i=0}^{t}\left(1-\beta_{t}\right)$, t∈(0,1,…,T−1) and $\epsilon_{\theta }\left(\cdot \right)$ is a noise predictor based on a time-conditional UNet [6]. For efficiency, DDIM is employed in this work to generate the watermarked images.

To enhance the robustness and reduce computational complexity, we employ the variational autoencoder to transform images into the latent distribution and apply sampling in latent space [20]. As the latent space is much smaller compared to the original image space, the denoising and its reverse process run much faster.

### 2.2. Watermark Embedding

The goal of our watermarking scheme is to enable the extraction of the embedded watermark under various attacks and to provide high fidelity of the original image. To achieve this goal, we generate random watermark information $M=[m_{0},m_{1},\ldots ,m_{k-1}]$, $m_{i}\in \left\{0,1\right\}$ and orthonormal code vectors $P_{i}=\left[p_{i0},p_{i1},\ldots ,p_{i(n-1)}\right]$, n=2k and i∈[0,k−1]. The modulation process for generating the spread spectrum watermark $W=[w_{0},w_{1},\ldots ,w_{n-1}]$ can be simplified to:

(4) $$w_{j}=\sum_{i=0}^{k-1}\left(\left(2m_{i}-1\right)\cdot p_{ij}\right),$$

where j=0,1,…,n−1. To embed the watermark W, the clean image $I_{\mathrm{ori}}\in R^{3\times W\times H}$ is mapped to the latent space using the VAE encoder to obtain the latent vector $z_{0}\in R^{4\times W/f\times H/f}$, where f is the scale factor set to 8 in this work. Estimated noise is applied to $z_{0}$ in T time steps, yielding $z_{T}\in R^{4\times W/f\times H/f}$:

(5) $$z_{t+1}=\sqrt{{\bar{\alpha }}_{t+1}}{\hat{z}}_{0}^{t}+\sqrt{1-{\bar{\alpha }}_{t+1}}\epsilon_{\theta }\left(z_{t}\right),$$

where ${\bar{\alpha }}_{t}=\prod_{i=0}^{t}\left(1-\beta_{t}\right)$ and $\epsilon_{\theta }\left(\cdot \right)$ is a noise estimator. Then, the noisy latent vector $z_{T}$ is split into four slices along the channel dimension. It is empirically found that embedding the watermark in the fourth channel leads to better image quality, in spite of the fact that the embedding capacity would profit from using all the four channels. Considering the invisibility of watermarks, watermark embedding is performed in 8 × 8 DCT blocks of $z_{T}$. We extract the seventh high-frequency coefficients on the main diagonal from each block and concatenate them to construct the embedding matrix $F_{\mathrm{co}}=\left[F_{0},F_{1},\ldots ,F_{n-1}\right]$. The watermark embedding is depicted as:

(6) $$F_{w}=F_{\mathrm{co}}+S\cdot W,$$

where S is the strength factor of the embedding. IDCT is applied to $F_{w}$ to update $z_{T}$ as $z_{T}^{\prime }$. Finally, the watermarked image $I_{\mathrm{gen}}$ is yielded by

(7) $$I_{gen}=Dec\left(\epsilon_{\theta }\left(z_{T}^{\prime },T\right)\right),$$

where Dec(·) is the VAE decoder to transform the latent vector back into image space.

To further improve the image quality, the original image is partially added to the generated image to obtain the watermarked image $I_{w}$. The enhancement is achieved by

(8) $$I_{w}=\lambda I_{ori}+\left(1-\lambda \right)I_{gen},$$

where λ is a weighting parameter.

### 2.3. Watermark Extraction

As the inverse operation of watermark embedding, watermark extraction is also conducted in the noisy latent space. Therefore, the VAE encoder, as in the embedding process, is imposed on the watermarked image followed by the inversion process, yielding the latent vector $z_{\mathrm{Tw}}$. Then, we apply DCT on the channel that was selected for watermark embedding. The extracted DCT coefficients are denoted as $F_{w}^{\prime }=\left[F_{0}^{\prime },F_{1}^{\prime },\ldots ,F_{n-1}^{\prime }\right]$. Note that the indexes of DCT coefficients in $F_{w}^{\prime }$ are the same as that of the embedding. The original watermark is recovered by calculating the correlation between the spread spectrum code and the detection matrix:

(9) $$m_{i}^{\prime }=\left\{\begin{array}{cc} 1, & \mathrm{if}\;\langle F_{w}^{\prime },P_{i}\rangle \geq 0,\\ 0, & \mathrm{otherwise}, \end{array}\right.$$

where 〈·,·〉 denotes the inner product operation.

### 2.4. Loss Function

Although watermarking is performed in the noisy latent space, the loss of image quality is still inevitable. In order to minimize the loss of image quality, the mean squared error (MSE) between $I_{\mathrm{ori}}$ and $I_{\mathrm{gen}}$ is used to optimize the trainable latent vector:

(10) $$L_{I}=\frac{1}{CHW}{∥I_{ori}-I_{gen}∥}_{2}^{2}\;,$$

where C,H,W are the image channels, height, and width. Additionally, SSIM loss [21] is also included to achieve structural similarity to the original image

(11) $$L_{S}=1-\mathrm{SSIM}\left(I_{\mathrm{ori}},I_{\mathrm{gen}}\right).$$

To get closer to human perception, perceptual loss [22] is also included,

(12) $$L_{P}=\frac{1}{C_{j}H_{j}W_{j}}{∥\phi_{j}\left(I_{ori}\right)-\phi_{j}\left(I_{gen}\right)∥}_{2}^{2}\;,$$

where $\phi_{j}\left(\cdot \right)$ represents the output of the j-th layer of a pre-trained VGG model [23], $C_{j},H_{j},W_{j}$ are the number of channels, height, and width of the feature map in the j-th layer. The total loss is formulated as

(13) $$L=\gamma_{1}L_{I}+\gamma_{2}L_{S}+\gamma_{3}L_{P},$$

where $\gamma_{1}$, $\gamma_{2}$, and $\gamma_{3}$ are weight factors.

## 3. Experiments

### 3.1. Experimental Settings

To evaluate our scheme, we adopt two public datasets, i.e., MS-COCO [24] and DiffusionDB [25]. We randomly sample 200 images sized 512×512 from each dataset. The pre-trained stable diffusion model v2.1 [20] and the DDIM sampler [19] with a sampling time step of 50 are employed as the generator of watermarked images. For the weight factors of the loss function, $\gamma_{1}$, $\gamma_{2}$ and $\gamma_{3}$ are set to 10, 1, and 0.1, respectively. The Adam optimizer [26] is employed with an initial learning rate of $10^{-2}$ for 30 epochs. The learning rate is then decayed at a rate of 0.3 at the 31st and the 81st epoch. The framework is implemented by PyTorch 2.0.1 and trained on a single NVIDIA RTX 4080. The inference time of our model for watermarking an image is 404.87 s, and the time cost for watermark extraction is about 2.5 s per image. As robustness is one of the primary goals of watermarking techniques, the bit error rate (BER) is employed to evaluate the robustness of watermarks under various attacks. Some common attacks are considered, e.g., brightness or contrast adjustment, JPEG compression (Q = 50), Gaussian noise with σ = 0.05, blur with a filter size of 5, crop with a ratio of 0.5, and BM3D denoising [27]. Considering the latest attacks from deep generative models, VAE-based image compression with Q = 3 [28,29] and regeneration attack [16] are launched. For imperceptibility evaluation, we choose three metrics, i.e., PSNR, SSIM, and LPIPS [30]. Comparisons are made with four state-of-the-art (SOTA) deep learning-based methods HiDDeN [4], Stegastamp [5], SSL [10], and Stable Signature [12]. All methods set the watermark bit length of 32, except that stable signature uses the pre-trained model for 48 bits.

### 3.2. Imperceptibility Test

**Figure 3:**
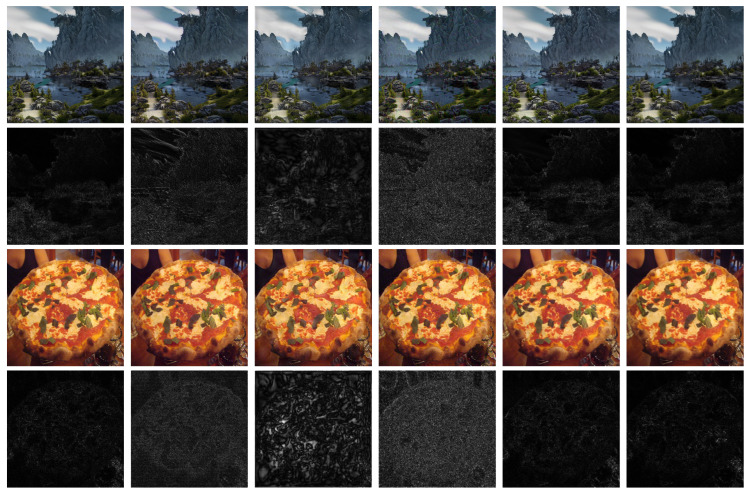
Visual quality comparison with various methods. From left to right are clean images, HiDDeN, Stegastamp, SSL, Stable Signature, and proposed. The second and fourth rows are the residual images.

Table 1 presents the comparison of image quality with various schemes derived from deep learning models, while Figure 3 displays the visual comparisons. As Table 1 and Figure 3 show, HiDDeN performs the best in terms of overall image quality. However, it is very limited in terms of robustness, as shown in Table 2, especially when facing attacks with global modification. For our method, the loss of image quality is mainly due to the reduced dimension of the latent space, while it is still comparable to other deep learning watermarking schemes. In Figure 3, it can be seen that the proposed method embeds watermarks in the edge details of images, thus leading to better imperceptibility. Additionally, the proposed method modifies fewer pixels of the original image, as illustrated by the comparison of residual images in Figure 3. In contrast to Stable Signature, which is also based on the diffusion model, our training strategy in the noisy latent space achieves less reconstruction loss and thus leads to better quality of watermarked images in terms of all the three metrics.

### 3.3. Robustness Test

**Figure 4:**
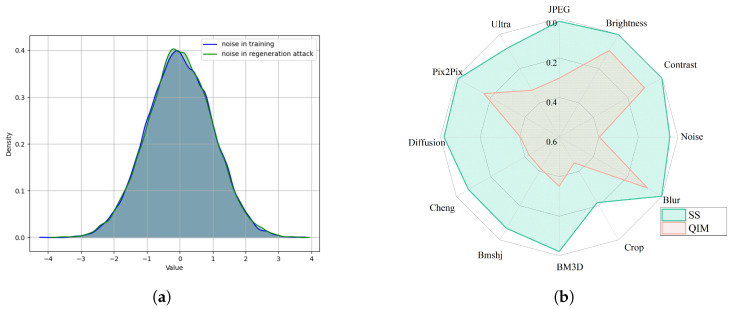
(**a**) Comparison of noise distribution; and (**b**) Visualization of the robustness comparison between using spread spectrum and QIM for watermarking.

It is observed in Table 2 that most of the methods are vulnerable to the regeneration attack, whereas our proposed scheme performs robustly under all the three regeneration attacks. Figure 4a visualizes the noise distribution introduced by the diffusion attack in latent space. It is seen that its distribution closely resembles the noise distribution used in training, and hence, the impact of this attack on our proposed scheme is negligible. Benefiting from this embedding process, the proposed method performs better in terms of generalization against regeneration attacks compared to those based on noise layer training. To verify the effectiveness of the spread spectrum scheme, the radar chart in Figure 4b compares the robustness of using QIM [31] as the embedding scheme, with the quantization step set to 50. The chart highlights the superiority of the spread spectrum watermarking in latent space.

**Table 2:** Performance comparison of robustness under different attacks. The numbers 1 to 7 represent JPEG, brightness, contrast, Gaussian noise, blur, crop, and bm3d attacks, respectively. And the abbreviations B, C, D, P, and U stand for Bmshj [28], Cheng [29], Diffusion [16], Instruct-Pix2Pix [32], and UltraEdit [33], respectively. The best result of each column is marked in **bold**, while the second-best is underlined.

Methods	Common Attacks							Regeneration Attacks			Image Editing	
	1	2	3	4	5	6	7	B	C	D	P	U
COCO												
HiDDeN	0.310	0.151	0.154	0.223	0.188	0.182	0.295	0.417	0.378	0.416	0.461	0.457
Stegastamp	**0.001**	**0.001**	0.002	**0.003**	**0.001**	0.186	**0.001**	**0.002**	**0.002**	0.137	0.058	0.200
SSL	0.032	**0.001**	**0.001**	0.036	**0.001**	0.201	0.122	0.318	0.290	0.207	0.131	0.409
Stable Signature	0.124	0.006	0.008	0.025	0.066	**0.012**	0.169	0.377	0.284	0.522	0.511	0.583
Proposed	0.014	**0.001**	**0.001**	0.041	**0.001**	0.217	0.021	0.067	0.070	**0.018**	**0.010**	**0.079**
Proposed (64)	0.013	**0.001**	**0.001**	0.032	**0.001**	0.221	0.019	0.059	0.070	**0.013**	**0.014**	**0.063**
DiffusionDB												
HiDDeN	0.319	0.155	0.155	0.226	0.184	0.187	0.305	0.420	0.385	0.422	0.481	0.448
Stegastamp	**0.001**	0.002	0.003	**0.003**	**0.001**	0.189	**0.001**	**0.002**	**0.003**	0.152	0.073	0.258
SSL	0.038	**0.001**	**0.001**	0.031	**0.001**	0.211	0.139	0.322	0.298	0.260	0.163	0.376
Stable Signature	0.113	0.005	0.006	0.013	0.062	**0.008**	0.146	0.375	0.275	0.521	0.482	0.552
Proposed	0.025	**0.001**	**0.001**	0.049	**0.001**	0.227	0.029	0.077	0.081	**0.028**	**0.018**	**0.100**
Proposed (64)	0.023	**0.001**	**0.001**	0.047	**0.001**	0.220	0.030	0.081	0.089	**0.018**	**0.025**	**0.094**

**Figure 5:**
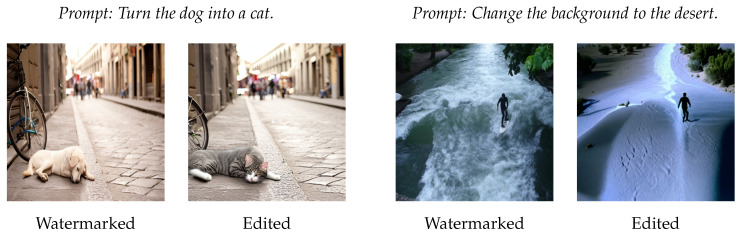
Performance of different prompts for image editing. The first group is the result with Instruct-Pix2Pix, the second is with UltraEdit.

To verify the effectiveness of the proposed under image editing attack, Instruct-Pix2Pix [32] and UltraEdit [33] are applied. The former is an instruction-driven image editing framework, enabling precise image modifications with user-specified commands, while the latter allows high-quality regional editing through the guidance of a mask. Figure 5 shows the editing styles imposed on the images, including local content replacement, background change and human action modification. These kinds of editing can modify the content of images easily and disrupt the embedded watermark information. The editing pipelines are controlled by the Text CFG (Classifier-free Guidance) and the Image CFG, which are the parameters for adjusting the relevance of the generated image to the prompt. In our experiment, the text and image CFG are set to 7.5 and 1.5, respectively. As demonstrated by the results shown in Table 2, the proposed method performs robustly to all kinds of image editing except image cropping. Although Stegastamp also shows very competitive results in terms of robustness to the attacks launched by generative models, our scheme excels a lot in terms of imperceptibility.

### 3.4. Capacity

In conventional watermarking systems, the embedding capacity is positively correlated with the size of the cover image. In contrast, our proposed method takes the latent representation of an image to hold the watermark. Since it is a low-dimensional representation of the image, it is much smaller in size than the cover image, limiting the embedding capacity of the watermark. On the flip side, embedding watermarks in the latent space ensures the robustness of watermarking systems, since transmission distortions and regeneration attacks tend to affect the high-frequency components of the image. To further evaluate the impact of the increased embedding capacity, we employ watermarks of 64 and 96 bits for the test. For 64 bits, the sixth coefficient on the main diagonal of each DCT block is additionally chosen for watermark embedding while the eighth coefficient is additionally selected for 96-bit embedding. From the results in Table 3, it is observed that a higher capacity like 96 bits leads to a significant degradation of image quality, in spite of a robustness gain as the reward. However, the watermarked image with an embedding capacity of 64 only suffers from slight image degradation, suggesting the trade-off between embedding capacity and imperceptibility of watermarks with our proposed scheme.

### 3.5. Discussions

**Figure 6:**
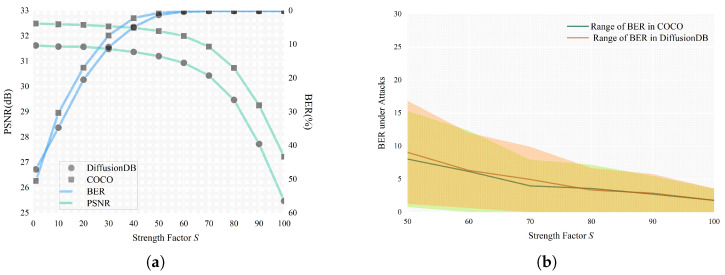
(**a**) PSNR and BER under different strength factors; (**b**) Range of BER under different attacks.

In this section, we discuss some of the key parameters that affect the robustness and imperceptibility of the proposed watermarking scheme. In traditional watermarking schemes, selecting an appropriate strength factor to balance the invisibility and the robustness of the watermark is a vital concern. Generally, a larger strength factor would lead to better robustness, while compromising the image quality. In Figure 6a, the impact of the strength factor on image quality and watermark robustness is plotted, measured in terms of PSNR and BER. As Figure 6a suggests, the proposed scheme is fragile given a mild strength factor of less than 50, even when no attack is imposed. This is because watermark embedding is performed in the noisy latent space and the noise predictor might be inaccurate when the amplitude is small. Drawing on this, Figure 6b presents the range of BER under different attacks, by applying the strength factor from 50 to 100. It is demonstrated that an optimal option exists at S = 70 since S larger than 70 can bring about severe image degradation. Considering the available editing techniques and the potential distortions brought by channel transmission, we prefer to have higher priority on robustness of the watermark given an acceptable quality of the watermarked image.

**Figure 7:**
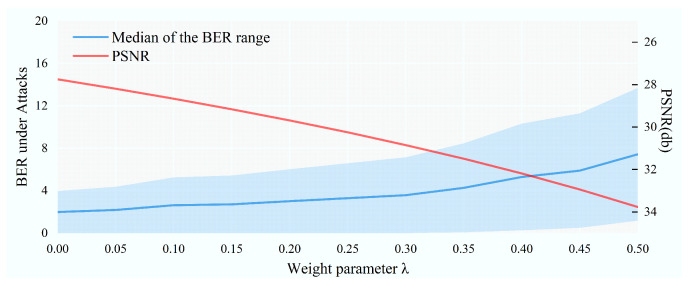
Parameter analysis about λ.

In addition, the parameter λ in Equation (8) controls the weight of raw data to further enhance image quality. Meanwhile, λ also affects the robustness of the watermark, so we sample at equal intervals in the range of [0,0.5] to determine the choice of λ. From Figure 7, a value of 0.3 is opted for considering the tradeoff between image quality and robustness against attacks.

## 4. Conclusions

In this paper, we propose an image watermarking framework that achieves spread spectrum watermarking in latent diffusion models, thus guaranteeing watermark imperceptibility and robustness. Essentially, the proposed design delivers comparable visual quality to the state-of-the-art methods, but outperforms in terms of resisting most recent attacks stemming from generative models like image editing and regeneration. In future work, we will further address the challenge from image cropping, rotation, and explore solutions for improving the watermark capacity in diffusion models.

## Figures and Tables

**Table 1:** Performance comparison of quantitative image quality among different methods. The best result is marked in **bold**, while the second-best is underlined.

	COCO			DiffusionDB		
Methods	PSNR ↑	SSIM ↑	LPIPS ↓	PSNR ↑	SSIM ↑	LPIPS ↓
HiDDeN	31.70	**0.93**	**0.02**	31.42	**0.94**	**0.02**
Stegastamp	28.73	0.89	0.07	28.05	0.89	0.07
SSL	**32.07**	0.87	0.11	**32.11**	0.88	0.10
Stable Signature	26.43	0.75	0.06	25.78	0.75	0.06
Proposed	30.85	0.90	0.05	30.49	0.90	0.05
Proposed (64)	28.28	0.88	0.10	28.63	0.89	0.09

**Table 3:** Performance comparison of the quantitative image quality and robustness under different embedding capacities in COCO dataset. The average BER of the three categories of attacks in Table 2 is computed to reflect the robustness. The best result is marked in **bold**.

		Transparency			Robustness		
	Capacity	PSNR ↑	SSIM ↑	LPIPS ↓	Common	Regeneration	Editing
	32	**30.85**	**0.90**	**0.05**	0.042	0.052	0.045
Proposed	64	28.28	0.88	0.10	0.041	0.071	0.039
	96	22.73	0.78	0.27	**0.026**	**0.022**	**0.026**

## Data Availability

The data presented in this study are available on request from the corresponding author.
